# Consensus Among Differential Item Functioning Effect Size Measures: A Simplified Approach to Reporting Effect Size

**DOI:** 10.1177/00131644261458305

**Published:** 2026-07-15

**Authors:** Austin Wyman, Zhiyong Zhang

**Affiliations:** 1 Department of Psychology, University of Notre Dame, IN, USA

**Keywords:** differential item functioning, effect size, measurement bias, item response theory, polytomous

## Abstract

Differential item functioning (DIF) is an issue of a measure that can lead to differences in item scores between subpopulations (e.g., sex, race, age) while controlling for a latent trait or ability level. It is important to report DIF effect size measures to understand the practical significance of DIF results as well. We reviewed 15 different DIF effect size measures in the literature, and no previous study had examined whether the 15 DIF effect size measures agreed with one another, which has implications in the number and type of measures to report. The present study investigated the relationship among DIF effect size measures in 1PL, 2PL, and GRM models and provide clear recommendations to applied researchers for their reporting. Our simulation results showed that, for each model, three effect size measures can be reported to obtain the most unique information about uniform DIF and the least bias from sampling conditions (e.g., sample size and impact): Mantel-Haenszel Delta, McFadden’s Pseudo R-squared, and a signed DIF measure. However, the optimal signed DIF measure varies by model, with Signed Area under the curve for the 1PL model, Standardized P-difference for the 2PL, and Signed Item Difference in the Sample/Normal Distribution for the GRM. The recommended approach to reporting DIF effect size improves the detection of DIF through practical significance and provides researchers with more tools to address bias.

## Introduction

### Measurement Bias and Differential Item Functioning

Measurement bias is defined as cases in which measurement of an observed variable has violated the assumption of unobserved conditional variance. The assumption posits that the conditional probability of an observed response given a latent variable should equal the conditional probability of an observed response given a latent variable and some observed demographic variable, such as race, sex, or socioeconomic status (Millsap & Everson, 1993). When the assumption is violated, it indicates a portion of the observed is being explained by a factor other than the latent variable, which constitutes bias. Measurement bias can be identified at multiple levels of measurement, but there are distinct advantages to studying bias at the item level. Item-level analysis is more precise than test- or scale-level analysis, providing greater sensitivity for the detection of bias (Zumbo, 2003), greater interpretability for the mechanism of bias (Cheng et al., 2016), and greater actionability for addressing bias once it is discovered. At the item level, a framework for detecting bias is known as Differential Item Functioning (DIF).

DIF describes cases in which there is a difference in observed item scores between two subpopulations (e.g., race, ethnicity, gender, age, socioeconomic status) while controlling for ability level. When DIF is present, individuals with the same latent trait level but belonging to different groups, such as gender, would receive different observed scores. One example from clinical psychology is that an assessment for autism would rate males as having higher empathy than females despite having the same autistic trait level (Yu et al., 2026). DIF can be expressed mathematically as the following inequality,

(1) $$P\left(X_{\mathrm{ij}}=1|F,\theta \right)\;\neq \;P\left(X_{\mathrm{ij}}=1|R,\theta \right),$$

where *F* describes group membership in the focal group and *R* descries membership in the reference group. The equation relates to dichotomous item response data, but it also generalizes well to polytomous data.

There are two types of DIF: *uniform* DIF and *nonuniform* DIF. For uniform, the difference between the focal group and the reference group is constant for every level of ability (Hanson, 1998). Nonuniform DIF is observed when the difference between focal and reference groups is not constant across ability. It is important to note that DIF is distinct from the concept of *impact*. Impact refers to group differences between subpopulations, such that the ability of one group of respondents is a constant higher than the ability of another group. DIF and impact both describe differences between subpopulations, but the important distinction between the two is that DIF removes the influence of ability level, while ability level is the main feature of impact.

There are numerous statistical methods, both parametric (e.g., likelihood ratio test, logistic regression) and nonparametric (e.g., Mantel–Haenszel test, simultaneous item bias test), to detect DIF within psychological and educational measurement, and each approach has distinct advantages and disadvantages. The lack of a consistent direction for researchers to conduct DIF analysis is problematic because different DIF methods perform differently—particularly with respect to Type I error control and statistical power—under diverse circumstances (often relating to data type), and using the wrong method for the wrong data can result in falsely flagging items as having DIF (i.e., Type I error) or falsely claiming that an item is free of DIF (i.e., Type II error).

### Detecting DIF Through Effect Size

Statistical tests can be powerful ways to detect DIF in a measure, and they are certainly the most popular approach. However, the use of statistical tests for DIF detection introduces multiple limitations. Statistical tests are a function of sample size, and as sample size becomes increasingly large, the statistical test will converge toward a significant result (Shaver, 1993). Meehl (1967) argued that any statistical test, no matter how trivial, will eventually become statistically significant at high-enough sample sizes. This is especially challenging for education research, where high-stake assessments (e.g., Graduate Record Examination) can contain thousands of respondents. It is also challenging to interpret statistical test results beyond the binary of significant and nonsignificant. When two or more items emerge as significant, there is no framework within statistical testing that establishes how to compare significant results or rank their importance. Therefore, it is important to differentiate the practical significance of DIF from the statistical significance of a DIF test.

Kelley and Preacher (2012) define effect size as “a statistic (or parameter) with a purpose, which is to quantify some phenomenon that addresses a question of interest.” Effect sizes often accompany statistical tests as a clarification of the test result but can also be used independently. Effect sizes are typically designed to be independent of sample size (Shaver, 1993). Therefore, effect sizes are valuable tools for interpreting the practical significance of a result, by removing spurious factors that may influence statistical significance.

Many DIF effect size measures have been proposed. A comprehensive literature review of DIF effect size measures, their formulations, and strengths and weaknesses can be found in the Supplemental Material. In summary, we reviewed and identified 15 DIF effect size measures that can be used appropriately in both dichotomous and polytomous Item Response Theory (IRT) models. These measures may further be classified into signed and unsigned DIF measures. Signed measures refer to DIF effect size measures that communicate the directionality of DIF (i.e., whether DIF favors the reference group or focal group). Unsigned measures refer to DIF effect size measures that only communicate the magnitude of DIF, often involving taking the squared difference between groups or the absolute value of differences. These effect size measures were the focus of the present paper, and they are summarized in the following Tables 1 and 2.

**Table 1.** Summary of Signed DIF Effect Size Measures.

Metric	Equation	Citation
Mantel–Haenszel Common Odds Ratio (MH Alpha)	${\hat{\alpha }}_{\mathrm{MH}}=\;\frac{\sum_{j}A_{j}D_{j}/N_{j}}{\sum_{j}B_{j}C_{j}/N_{j}}$	Holland and Thayer (1986)
Mantel–Haenszel Delta (MH Delta)	${\hat{\Delta }}_{\mathrm{MH}}=-2.35\;\ln\left({\hat{\alpha }}_{\mathrm{MH}}\right)$	Holland and Thayer (1986)
Mantel Cumulative Odds Ratio (Polytomous)	${\hat{\alpha }}_{\mathrm{Mantel}}=\frac{\sum_{k=1}^{K}\sum_{j=1}^{c-1}X_{1\mathrm{jk}}^{*}(n_{2k}-X_{2\mathrm{jk}}^{*})/N_{k}}{\sum_{k=1}^{K}\sum_{j=1}^{c-1}(n_{1k}-X_{1\mathrm{jk}}^{*})X_{2\mathrm{jk}}^{*}/N_{k}}$	Liu and Agresti (1996)
Standardized P-Difference (STD)	$\mathrm{STD}=\sum_{s}k_{s}\left(P_{\mathrm{Fs}}-P_{\mathrm{Rs}}\right)/\sum_{s}k_{s}\;$	Dorans and Kulick (1983)
Signed Item Difference in the Sample (SIDS)	${\mathrm{SIDS}}_{j}=\;\sum_{i=1}^{N}\left[{\mathrm{ES}}_{\left(\mathrm{ij}|\hat{\theta },\gamma_{F}\right)}\;-{\mathrm{ES}}_{\left(\mathrm{ij}|\hat{\theta },\gamma_{R}\right)}\right]/N$	Meade (2010)
Signed Item Difference in the Normal Distribution (SIDN)	${\mathrm{SIDN}}_{j}=\sum_{\theta }\left[{\mathrm{ES}}_{\theta ,\gamma F}-{\mathrm{ES}}_{\theta ,\gamma R}\right]w_{\theta }$	Meade (2010)
Maximum Difference in the Sample (D-Max)	$D-\mathrm{Max}\;=\;\mathrm{argmax}\left[{\mathrm{ES}}_{\left(\mathrm{ij}|\hat{\theta },\gamma_{F}\right)}\;-{\mathrm{ES}}_{\left(\mathrm{ij}|\hat{\theta },\gamma_{R}\right)}\right]$	Meade (2010)
Expected Score Standardized Difference (ESSD)	$\mathrm{ESSD}\;=\;\frac{{\bar{\mathrm{ES}}}_{\left(\gamma F\right)}-{\bar{\mathrm{ES}}}_{\left(\gamma R\right)}}{{\mathrm{SD}}_{\mathrm{Pooled}}}\;$	Meade (2010)
Compensatory DIF (CDIF)	${\mathrm{CDIF}}_{j}=\mathrm{Cov}\left(d_{i},D\right)+\mu_{d_{j}}\mu_{D}$	Raju et al. (1995)
Raju’s Signed Area (SA)	$\mathrm{SA}\;=\;\int_{-\infty }^{\infty }\left({\hat{F}}_{R}-{\hat{F}}_{F}\right)d\theta \;$	Raju (1990)
SA (Polytomous)	${\mathrm{SA}}_{\mathrm{GRM}}=\sum_{k=1}^{m-1}\left(u_{\left(k+1\right)}-u_{k}\right)\int_{-\infty }^{\infty }\left(P_{\mathrm{kR}}\left(\theta \right)-P_{\mathrm{kF}}\left(\theta \right)\right)d\theta \;$	Cohen and Kim (1993)

**Table 2.** Summary of Unsigned DIF Effect Size Measures.

Metric	Equation	Citation
Root Mean Weighted Standardized Difference (RMWSD)	$\mathrm{RMWSD}\;=\;\sqrt{\sum_{s}k_{s}{\left(P_{\mathrm{Fs}}-P_{\mathrm{Rs}}\right)}^{2}/\sum_{s}k_{s}\;}$	Dorans and Kulick (1983)
Unsigned Item Difference in the Sample (UIDS)	${\mathrm{UIDS}}_{j}=\sum_{i=1}^{N}\left|{\mathrm{ES}}_{\left(\mathrm{ij}|\hat{\theta },\gamma_{F}\right)}\;-{\mathrm{ES}}_{\left(\mathrm{ij}|\hat{\theta },\gamma_{R}\right)}\right|/N$	Meade (2010)
Unsigned Item Difference in the Normal Distribution (UIDN)	${\mathrm{UIDN}}_{j}=\sum_{\theta }\left[\left|{\mathrm{ES}}_{\theta ,\gamma F}-{\mathrm{ES}}_{\theta ,\gamma R}\right|\right]w_{\theta }$	Meade (2010)
McFadden’s Pseudo R-squared	$R_{\mathrm{MF}}^{2}=1-\frac{{\mathrm{LL}}_{M}}{{\mathrm{LL}}_{0}}\;$	McFadden (1974)
Noncompensatory DIF (NCDIF)	${\mathrm{NCDIF}}_{j}=\sigma_{d_{j}}^{2}+\mu_{d_{j}}\;$	Raju et al. (1995)
Raju’s Unsigned Area (UA)	$\mathrm{UA}\;=\;\int_{-\infty }^{\infty }\left|{\hat{F}}_{R}-{\hat{F}}_{F}\right|d\theta \;$	Raju (1990)
UA (Polytomous)	${\mathrm{UA}}_{\mathrm{GRM}}=\sum_{k=1}^{m-1}\left(u_{\left(k+1\right)}-u_{k}\right)\int_{-\infty }^{\infty }\left|P_{\mathrm{kR}}\left(\theta \right)-P_{\mathrm{kF}}\left(\theta \right)\right|d\theta \;$	Cohen and Kim (1993)

### Barriers to Interpreting DIF Effect Size

DIF effect sizes are intended to be interpretable representations of DIF effects, but their interpretability depends on multiple factors. All DIF effect size measures can easily be interpreted comparatively (i.e., across different results). For example, if a study reports the Standardized P-Difference (STD) for two items, it is easy to determine that the item with the higher STD value has the higher magnitude of DIF. However, it is much more challenging to interpret effect sizes in isolation. Without another value to compare against, obtaining an STD value of 0.5 for an item is unintuitive. Comparisons are also only available when the same effect size metric is used. In studies where different effect size measures are used, the lack of a common scale makes it impossible for researchers to link results. For example, if one study only reports the Pseudo R^2^ for an item’s DIF effect size and another study only reports Unsigned Item Difference in the Sample (UIDS), it is impossible to tell which study’s DIF effect has greater magnitude. Despite the large number of DIF effect size measures, there is no standard practice for reporting effect sizes for DIF analysis, nor is there clarification regarding which effect size to select. Moreover, Kim and Cohen (1995) argue that there is little evidence that any one DIF detection method is superior, and they encourage researchers to use multiple detection approaches. Hence, the problem of encountering multiple effect size measures and communicating across scales in inescapable. The lack of a valid scale for comparison silos DIF studies and harms scientific replication.

As a consequence of poor guidelines, DIF effect size measures are rarely reported in empirical literature. In May 2026, a crude estimate of the number of publications including DIF effect size measures was obtained using Google Scholar by searching specific DIF effect size measures, including empirical study terms (e.g., “participants,”“sample,”“dataset”) and excluding common simulation study terms (e.g., “methodological,”“conditions under,”“parameter recovery”). The search containing effect sizes resulted in 34,400 publications. Of course, this number is contrasted with the total number of empirical DIF publications, which, through a similar google scholar search approach, is estimated to be about 2,110,000. Therefore, approximately 1.6% of DIF studies in the literature report DIF effect size measures.

Better interpretation guidelines for DIF effect size measures requires understanding the relationship between measures. Simulation studies have examined agreement between specific effect size indices, often within classes of the same method. For example, Fleer (1993) found that noncompensatory DIF (NCDIF), Raju’s Signed Area (SA), and Raju’s Unsigned Area (UA) correlated strongly with each other, which is expected given that each method is parametric, follows the same IRT model, and evaluates differences in item response functions. Although work has been conducted within classes of methods, there has been no holistic study, particularly across classes, that examines the agreement between DIF effect size measures, which poses a significant barrier for their interpretation. Interpretative guidelines would greatly improve interpretations of effect size measures independently but have the potential to bridge interpretations between effect size measures as well through the use of a common scaling variable, which would link the different measures to the same units.

### Current Study

The purpose of the present study is to understand the relationship among different DIF effect size measures and the conditions under which effect size estimates may fluctuate. Through Monte Carlo simulation, the present study is able to specify the DIF effect size in the population (i.e., difference between population IRFs) and measure the extent to which DIF effect size measures accurately estimate the true effect. The effect sizes that the study evaluated are comprehensive, including the MH Alpha, MH Delta, STD, RMWSD, SA, UA, CDIF, NCDIF, SIDS, UIDS, Signed Item Difference in the Normal Distribution (SIDN), Unsigned Item Difference in the Normal Distribution (UIDN), Expected Score Standardized Difference (ESSD), Maximum Difference in the Sample (D-Max), and Pseudo R-squared. The study is exploratory in nature. It contributes to DIF literature by being the first study to evaluate 15 different DIF effect size measures and describe their relationship. The study is also the first to postulate what constitutes a “good” effect size measure for DIF and provides clear recommendations for reporting effect sizes, which may improve the accessibility of DIF analysis and encourage more scientists to report effect size.

## Method

A Monte Carlo simulation study was conducted to examine the agreement among DIF effect size measures under different IRT models and common data conditions. Data was simulated from 1PL, 2PL, and GRM models, which are the most-used IRT models in the social and behavioral sciences. Table 3 provides a complete list of the factors used to design the simulation study, including sample size, size of uniform DIF, total number of items, proportion of DIF items, proportion of focal group, size of impact, size of nonuniform DIF, and number of item response categories. The sample sizes were selected according to Svetina Valdivia and Dai’s (2024) minimum sample size for GRMs at 250 respondents and Jiang et al.’s (2016) upper asymptote at 1,000 respondents. Uniform DIF effect size was set to range from 0 to 2, increasing in increments by 0.25. The total number of items was either 10, representing a brief measure, or 25, representing a longer measure. The proportion of items that contain DIF was either 0.1 or 0.2. The proportion of the total sample size that is a member of the focal group was 0.1, 0.2, or 0.5, since practically focal groups in DIF studies are either balanced or minority sampled. The size of impact was 0 (indicating no impact), 0.5 (medium impact), or 1 (large impact). The size of nonuniform DIF follows the same scale as impact but was confined to 2PL and GRM—models that possess a discrimination parameter. Finally, the total number of item response categories was 2 (i.e., binary items), 3 (i.e., 3-point Likert-type scale), or 5 (i.e., 5-point Likert-type scale). The number of categories was held constant for simulations involving dichotomous items but was varied for simulations involving the GRM. All other conditions were varied during simulations with all three models. One thousand repetitions were conducted for every simulation condition.

**Table 3.** Summary of Simulation Conditions Across All Three Population Models.

Condition	Level	Model
Sample size	*N* = {250, 500, 750, 1000}	1PL, 2PL, GRM
Uniform DIF effect size	*D* = {0, 0.25, 0.50, 0.75, 1.00, 1.25, 1.50, 1.75, 2.00}	1PL, 2PL, GRM
Total number of items	*J* = {10, 25}	1PL, 2PL, GRM
Proportion of DIF items	*Nd* = {0.1, 0.2}	1PL, 2PL, GRM
Proportion of focal group	*Nf* = {0.1, 0.2, 0.5}	1PL, 2PL, GRM
Size of impact	*I* = {0, 0.5, 1}	1PL, 2PL, GRM
Size of nonuniform DIF	*Nu* = {0, 0.5, 1}	2PL, GRM
Number of response categories	*K* = {3, 5}	GRM

All item response data were simulated using the simdata function from the multidimensional item response theory (mirt) package in *R* (Chalmers, 2012). The function requires four inputs: the discrimination parameter (α), difficulty parameter (β), ability parameter (θ), and item type, which refers to the IRT model that it follows. θ was generated identically across the three IRT models to follow a standard normal distribution. θ was generated separately for the reference group and focal group. In conditions where there is an impact, θ for the focal group was generated with a mean lower than that of the reference group, such as 0.5.

β was generated similarly in the 1PL and 2PL model. β was a J × 1 vector, where J is the total number of items, and each β followed a uniform distribution, where the minimum is 0.5 and the maximum is 2. However, for the GRM, β was specified as a J × (K – 1) matrix, where K is the number of item response categories. The first threshold of β was generated separately and followed a uniform distribution, ranging from 1/K to K. Each subsequent threshold parameter was generated by creating a new value from the uniform distribution, with 1/K minimum and 3 – 1/k maximum, and subtracting the new value from the previous threshold parameter. This ensured relatively stable increases in β across thresholds for each item. Across all three models, β was generated separately for the reference group and focal group. β for the reference group was generated normally, but β for the focal group was a constant smaller than the reference group to introduce DIF. The location of DIF is supported by Raju’s (1990) definition of the exact signed area, which is described as the difference in difficulty parameters between the reference and focal groups. The definition is standard practice for incorporating DIF into simulated response data and has been implemented in many simulation studies of DIF (Fidalgo et al., 2004). The size of the β difference was determined by the uniform DIF effect size simulation parameter.

In models where the α parameter is available (2PL and GRM), α was identically generated across the models to follow a truncated normal distribution, in which the lower bound is 0. The mean and standard deviation of the distribution were sampled from a uniform distribution of α parameters, ranging from .5 to 2 to sample α parameters that closely resembled real data. In cases where there was nonuniform DIF, α was generated normally for the reference group, but α for the focal group was a constant smaller. The size of the constant is determined by the nonuniform DIF size parameter, which is distinct from the uniform DIF effect size parameter. Nonuniform DIF effect size was not analyzed in the present study; however, it is an important condition to understand modeling situations, which may influence DIF effect size measures to disagree with each other.

The 15 DIF effect size measures were obtained using a variety of IRT packages in R. SIDS, UIDS, SIDN, UIDN, ESSD, and D-Max were obtained from the mirt package after fitting a likelihood ratio test to the 1PL, 2PL, and GRM models. SA, UA, CDIF, and NCDIF were obtained from the DFIT package (Cervantes, 2017), which extracted parameters from the preceding mirt model. MH Alpha, MH Delta, and the corresponding MH cumulative odds ratio corresponding to the polytomous case were obtained from the difR package (Magis et al., 2010). MH Delta for the polytomous case was computed manually from the polytomous MH Alpha estimate using the transformation in Equation (3). STD was also obtained through a difR function, but the function was modified to allow for the computation of Root Mean Weighted Standardized Difference (RMWSD) as well. Finally, the logistic regression approach was conducted, and Pseudo R-squared was obtained using a modified function of difR.

Two analyses were conducted on the simulated data set to answer the study’s two main research questions. First, correlations among DIF effect size measures were examined to assess the convergence among different methods. Pearson’s and Spearman’s correlations were both computed as the present study was agnostic as to whether the relationship among measures would be linear or monotonic. Correlations were computed within each model type separately across all simulation conditions. Next, linear regression models were also conducted with DIF effect size measures as the dependent variable and simulation conditions as the independent variables to understand the sampling and population conditions in which effect size measures may fluctuate. Standardized coefficients were reported in regression analysis.

## Results

All 1,296 conditions in the 1PL model were successfully completed, as did the 3,888 conditions of the 2PL model and 7,776 conditions of the GRM. The total number of replications was 3,240,000 for the 1PL model, 9,720,000 for the 2PL, and 18,136,000 for the GRM. Visual inspection of the effect sizes identified some outliers, less than 1% of all replications, which were removed prior to the analysis. The extreme effect size values arose from the formulation of the effect sizes themselves.

### Correlations Among DIF Effect Size Measures

#### 1PL Model

Table 4 presents the Pearson’s correlations of the effect size measures. Spearman’s correlations may be found in Table S1 of the Supplemental Material. In both correlation matrices, the SIDS, UIDS, SIDN, and UIDN were perfectly or near-perfectly correlated with one another. These measures belong to the same class of effect size measures—the expected score-based measures—and so it is expected that they would be closely related to each other. The difference between signed and unsigned effect size measures is intended to account for crossing signs, which can be attributed to nonuniform DIF. The 1PL model lacks a discrimination parameter, so the signed and unsigned measures are both influenced exclusively by the uniform DIF effect size in the population. Therefore, the SIDS and UIDS should be perfectly correlated with one another. The same relationship was observed for the SA and UA measures, which were near perfectly correlated with one another in the absence of any crossing signs.

**Table 4.** Pearson’s Correlation Matrix of DIF Effect Size Measures in 1PL Model.

	A	B	C	D	E	F	G	H	I	J	K	L	M	N	O
A	–														
B	.**99**	–													
C	**1**	.**99**	–												
D	.**99**	**1**	.**99**	–											
E	.**90**	.**89**	.**90**	.**89**	–										
F	.**86**	.**84**	.**88**	.**86**	.**87**	–									
G	.**56**	.**56**	.**57**	.**57**	.**62**	.**71**	–								
H	**–.68**	**–.67**	**–.70**	**–.68**	**–.72**	**–.82**	**–.91**	–							
I	.**82**	.**80**	.**83**	.**82**	.**86**	.**99**	.**75**	**–.84**	–						
J	.**81**	.**81**	.**82**	.**82**	.**85**	.**97**	.**76**	**–.83**	.**99**	–					
K	.**73**	.**74**	.**74**	.**75**	.**65**	.**68**	.29	–.35	.**67**	.**68**	–				
L	.**95**	.**96**	.**95**	.**96**	.**87**	.**81**	.**60**	**–.67**	.**79**	.**80**	.**77**	–			
M	.**81**	.**80**	.**81**	.**80**	.**72**	.**68**	.**68**	**–.82**	.**65**	.**63**	.38	.**78**	–		
N	.47	.47	.48	.49	.40	.49	.44	–.48	.47	.48	.48	.49	.**50**	–	
O	.**61**	.**62**	.**61**	.**62**	.**59**	.**60**	.**72**	**–.75**	.**60**	.**61**	.27	.**64**	.**76**	.49	–

*Note*. A = SIDS, B = UIDS, C = SIDN, D = UIDN, E = ESSD, F = D-max, G = MH Alpha, H = MH Delta, I = SA, J =UA, K = CDIF, L = NCDIF, M = STD, N = RMWSD, O = Pseudo R-squared. *r* >|.5| are bolded, and|.3| < *r* >|.5| are underlined.

Similarly, the sample-based measures (i.e., SIDS and UIDS) were perfectly correlated with the normal distribution-based measures (i.e., SIDN and UIDN). Normally, this would not be the case; however, the current simulation assumed that the theta followed a normal distribution, making the two vectors of theta equivalent to each other. Therefore, in the present study, SIDS and UIDS and SIDN and UIDN are equivalent to each other, but this may rarely be the case in real data. The other expected score-based measures—ESSD and D-Max—were less correlated than SIDS, UIDS, SIDN, and UIDN but still demonstrated a strong linear and monotonic relationship with all the other measures of the same class.

Although the STD and RMWSD share a lot of similarities with the expected score-based measures, notable differences in the correlation estimates were observed. STD and RMWSD are intended to be signed and unsigned counterparts of each other, yet the Pearson’s correlation between the two measures was only .50, and the Spearman’s correlation was .48. The weakened relationship suggests that either STD and RMWSD are measuring different aspects of uniform DIF effect size or that there are simulation conditions besides DIF effect size that are biasing the estimate of either metric or both. STD is strongly correlated with all the expected score-based measures, whereas RMSWD is moderately correlated with everything, even less than its relationship with STD. The lack of relationship with all other measures indicates that RMSWD is biased, and that STD, though attenuated, is functioning more properly.

Another pair of measures—CDIF and NCDIF—were strongly correlated with each other (Pearson’s *r* = .77 and Spearman’s *r* = .79). Although NCDIF is an unsigned counterpart of CDIF, the two measures are distinct even when nonuniform DIF is absent because CDIF measures uniform and compensatory DIF, whereas NCDIF measures uniform and nonuniform DIF. Even under completely uniform DIF conditions, compensatory DIF is present and influences CDIF away from NCDIF. NCDIF was strongly correlated with many other DIF effect size measures, particularly the expected score-based measures. Pseudo R-squared exhibited similar relationships; however, the metric was more correlated with Raju’s area methods.

The MH measures were correlated with most other DIF effect size measures, although correlations were low enough to suggest that the measures are distinct. MH Alpha and Delta are derived from nonparametric DIF-detection techniques, whereas the other methods are mostly used as companions to parametric methods. Hence, the MH have more in common with nonparametric measures like STD than the expected score-based measures. The exception to this is RMWSD, which, although nonparametric, is biased by simulation conditions. It is important to also acknowledge that MH Delta was the only metric that observed all negative correlations for both Pearson’s and Spearman’s correlations. MH Delta is interpreted so that positive values indicate an item is more difficult for the reference group, and negative values indicate it is more difficult for the focal group, which is the inverse of how other DIF effect size measures are interpreted, with positive values indicating higher difficulty for the focal group. Item response data was generated with DIF biasing the focal group for all items, so most DIF effect size measures are rated positively. Only MH Delta is rated negative consistently in the current data-generation procedure.

A linear regression model was fitted to each of the DIF effect size measures with the six simulation conditions as predictor variables. The standardized beta coefficients are presented in Table 5. The effect of sample size on effect size was negligible across the 15 measures, which is expected and a desired property.

**Table 5.** Standardized Regression Coefficients Predicting DIF Effect Size Measures With 1PL Simulation Conditions.

	*N*	*D*	*J*	*N*_*d*_	*N*_*f*_	*I*
SIDS	.026	.**632**	–.022	–.008	.022	.378
UIDS	.017	.**626**	–.023	–.009	.016	.372
SIDN	.025	.**643**	–.012	–.009	.021	.385
UIDN	.016	.**638**	–.013	–.009	.015	.379
ESSD	.011	.**667**	–.127	–.005	.007	.409
D-max	.044	.**743**	–.001	–.007	.038	.452
MH Alpha	–.033	.**706**	–.036	–.086	–.033	.013
MH Delta	–.005	**–.829**	.031	.073	.003	–.002
SA	.032	.**747**	–.012	–.007	.023	.456
UA	.023	.**746**	–.012	–.007	.017	.454
CDIF	.038	.358	.287	.054	.034	.**613**
NCDIF	.002	.**620**	–.011	–.008	.005	.369
STD	–.009	.**716**	.017	–.073	–.001	–.094
RMWSD	–.055	.471	.**546**	–.051	–.002	.077
R-squared	–.075	.**669**	–.017	–.096	.315	–.088

*Note*. *N* = sample size, *D* = uniform DIF effect size, *J* = number of items, *N*_*d*_ = number of DIF items, *N*_*f*_ = size of focal group, and *I* = size of impact. β coefficients >|.5| are bolded, and those between|.3| and|.49| are underlined.

A similarly small effect was observed for the size of the focal group on effect size, indicating most of the measures are not influenced by class imbalance between the reference and focal groups. However, focal group size did have a moderate effect on Pseudo R-square, which is expected because the Pseudo R-square measures are widely known to be biased by imbalanced data. That said, McFadden’s Pseudo R-square performs better than most Pseudo R-square measures, meaning we can expect the effect of imbalance on effect size values to be much worse if using another measure of the same class.

The effect of the number of total items and the number of DIF items was also observed to be negligible across the 15 measures. However, it is important to note that there was a large effect of the number of items on RMWSD. It is the only metric to exhibit a relationship above a .5 standard deviation increase due to the number of items, which could explain its lack of concordance with the other DIF effect size measures. Although the difference statistic is the same between STD and RMWSD, the statistic in RMSWD is squared, which increases at a much faster rate than STD as the number of items increases. That said, the number of DIF items does not influence effect size estimates for any of the DIF effect size measures.

The conditions that had the largest influence on effect size measures were the size of impact and the size of uniform DIF. Nine DIF effect size measures were moderately susceptible to impact, but CDIF was the only variable that was strongly influenced. Many of the measures susceptible to impact belonged to the expected score-based family of measures, in which the reliance on theta estimates may introduce bias due to impact. However, CDIF is distinct from this family of DIF measures. Its large susceptibility to impact likely comes from the fact that it contains two components that rely on theta: covariance of the difference statistic and the mean of the difference statistic. As impact increases in the population, CDIF is likely to be twice as influenced as effect size measures that only contain one theta component, such as expected score-based measures. As nonparametric methods that do not consider theta at all, MH Alpha and Delta were least susceptible to impact.

By the definition of effect size, it is expected that the size of uniform DIF should influence DIF effect size measures the most. In fact, the effect persisting while controlling for all other simulation conditions is a strong indication that DIF effect size measures are functioning as intended. All measures except CDIF and RMSWD observed a standard deviation increase greater than .6 as uniform DIF effect size increased. RMSWD only observed a .471 standard deviation increase for every unit increase in uniform DIF effect size, which is likely due to parsed influence from the total number of items. Similarly, CDIF exhibited a .358 standard deviation increase, which could be because of compensatory DIF influencing estimates alongside uniform DIF, or it could be due to the influence of impact. MH Alpha and Delta performed the best as indicators of the population effect size while limiting influence from sample size, class imbalance, size of impact, and other noise conditions.

#### 2PL Model

New patterns of correlations emerged within the 2PL model in contrast with the 1PL model. In the 1PL model, there was high corroboration between the Pearson’s and Spearman’s correlation estimates. This is not replicated in the 2PL model. Instead, a general pattern emerges that the monotonic relationship is slightly weaker than the linear relationship, shrinking Spearman’s correlations estimates toward 0. However, there are many exceptions to this rule, such as cases where the Spearman’s correlation is actually higher. These exceptions are scattered across the correlation matrices and often occur within the same rows or columns. Table 6 presents the respective correlation matrices for the 2PL model. Spearman’s correlations are also presented in the Supplemental Materials, in Table S2. Differences between Pearson’s and Spearman’s correlations were negligible, so the remainder of the section primarily discusses the Pearson’s correlation results.

**Table 6.** Pearson’s Correlation Matrix of DIF Effect Size Measures in the 2PL Model.

	A	B	C	D	E	F	G	H	I	J	K	L	M	N	O
A	–														
B	–.42	–													
C	**1**	–.42	–												
D	–.43	**1**	–.43	–											
E	.**94**	–.38	.**94**	–.38	–										
F	.**86**	–.31	.**86**	–.32	.**79**	–									
G	.**70**	–.09	.**70**	–.09	.**67**	.**66**	–								
H	**–.77**	.35	**–.77**	.36	**–.72**	**–.73**	**–.84**	–							
I	.**75**	–.29	.**76**	–.30	.**77**	.**81**	.**56**	**–.60**	–						
J	–.13	.37	–.14	.40	–.18	–.13	.06	.03	–.22	–					
K	.38	.42	.39	.42	.36	.35	.41	–.35	.30	.17	–				
L	–.42	.**87**	–.42	.**88**	–.36	–.36	–.10	.38	–.28	.38	.42	–			
M	.**85**	–.43	.**85**	–.43	.**79**	.**72**	.**80**	**–.89**	.**64**	–.06	.37	–.41	–		
N	–.35	.30	–.35	.31	–.28	–.37	–.28	.38	–.31	.17	–.10	.29	–.48	–	
O	**–.61**	.**50**	**–.61**	.**50**	**–.55**	**–.55**	–.49	.**78**	–.45	.14	–.21	.**52**	**–.72**	.46	–

*Note*. A = SIDS, B = UIDS, C = SIDN, D = UIDN, E = ESSD, F = D-max, G = MH Alpha, H = MH Delta, I = SA, J =UA, K = CDIF, L = NCDIF, M = STD, N = RMWSD, O = Pseudo R-squared. *r* >|.5| are bolded, and|.3| < *r* >|.5| are underlined.

The most dramatic difference in the correlation matrices is the emerging disagreement between signed and unsigned DIF effect size measures, which is caused by the presence of a discrimination parameter, permitting nonuniform DIF. For example, SIDS and UIDS (and SIDN and UIDN) were perfectly positively correlated in the 1PL model, but in the 2PL model, they are moderately negatively correlated (Pearson’s *r* = –.42 and Spearman’s *r* = –.38). As nonuniform DIF increases alongside uniform DIF, the overall DIF effect increases, and so do unsigned measures (e.g., UIDS and UIDN). However, the increase in nonuniform DIF also disrupts the signs of SIDS and SIDN, causing the signed measures to decrease. The presence of nonuniform DIF makes SIDS and SIDN perform more, whereas it is a distinct advantage for UIDS and UIDN. Therefore, the relationship between the measures is inverted. It is important to note that SIDS and SIDN (and UIDS and UIDN) remain perfectly correlated with each other, just as in the 1PL model, because no change to the distribution of theta has been made across the models. A similar relationship holds for the remaining expected score-based measures (i.e., ESSD and D-Max), in which they are strongly positively correlated with the signed DIF measures, but moderately negatively correlated with the unsigned measures.

Attenuated correlations with unsigned measures are a common pattern in the 2PL model, extending beyond the expected score-based measures. For example, Raju’s area methods used to be strongly correlated with SIDS, UIDS, SIDN, and UIDN. Although SA is still strongly positively correlated with SIDS and SIDN, UA is moderately negatively correlated with the two signed measures. It is expected that signed and unsigned measures of any family would disagree, yet it is surprising that unsigned measures would disagree with each other. UA exhibits only a moderate positive linear relationship with UIDS and UIDN. UA is also distinct from NCDIF and Pseudo R-squared—two other unsigned measures. That said, UIDS, UIDN, and NCDIF maintain a strong relationship; however, the presence of nonuniform DIF attenuates the relationship compared to the correlations of the 1PL model.

Under the 1PL model, most of the DIF effect size measures were correlated with most other measures, the exception being CDIF and RMWSD. This pattern is not replicated in the 2PL model. In fact, the only metric that is strongly correlated with other effect size measures is Pseudo R-squared, exhibiting strong correlations to both signed and unsigned measures. At the same time, Pseudo R-squared is not perfectly correlated with any other metric. In the 2PL model, R-squared corroborates findings from most signed and unsigned DIF effect size measures, while still contributing unique information about uniform and nonuniform DIF in the population.

A linear model was also fitted to the 2PL model results to predict effect size with seven simulation factors, including the size of nonuniform DIF. Their standardized beta coefficients are depicted in Table 7. Compared to the regression for the 1PL model, the absolute value of the beta coefficients decreased. Similar to the 1PL model, conditions such as sample size, focal group size, number of items, and number of IDF items had negligible influence on the effect size measures. Where relationships between these conditions and measures did exist previously, they were now attenuated, such as the relationship between number of items and RMWSD. The size of nonuniform DIF is a new variable that was introduced into the 2PL simulation, but did not have a meaningful influence on DIF effect size measures. Oddly, signed measures, which are not supposed to adjust for nonuniform DIF, exhibited larger influences from nonuniform DIF effect size than unsigned measures did. Overall, the effect of nonuniform DIF was negligible.

**Table 7.** Standardized Regression Coefficients Predicting DIF Effect Size Measures With 2PL Simulation Conditions.

	*N*	*D*_*U*_	*J*	*N*_*d*_	*N*_*f*_	*I*	*D*_*NU*_
SIDS	.043	**–.627**	–.012	.000	.040	.**511**	.192
UIDS	–.081	.302	–.023	–.001	–.066	–.161	.067
SIDN	.043	**–.629**	–.013	.001	.040	.**510**	.192
UIDN	–.083	.307	–.015	–.001	–.067	–.165	.065
ESSD	.040	**–.630**	–.003	.001	.035	.**501**	.241
D-max	.045	**–.522**	–.013	.001	.040	.407	.151
MH Alpha	–.036	**–.636**	.001	.029	–.040	.108	.165
MH Delta	–.008	.**717**	–.013	–.060	.006	–.094	–.160
SA	.034	–.485	–.012	.001	.033	.371	.189
UA	–.107	.159	–.031	–.001	–.067	–.085	–.034
CDIF	–.010	–.301	.117	.076	–.003	.126	.189
NCDIF	–.101	.276	–.016	–.001	–.080	–.147	.056
STD	.007	**–.720**	–.015	.060	.001	.125	.218
RMWSD	–.080	.313	.487	–.061	–.037	.130	–.056
R-squared	–.056	.**578**	.005	–.077	.273	–.058	–.081

*Note*. *N* = sample size, *D*_*U*_ = uniform DIF effect size, *J* = number of items, *N*_*d*_ = number of DIF items, *N*_*f*_ = size of focal group, *I* = size of impact, and *D*_*NU*_ = nonuniform DIF effect size. β coefficients >|.5| are bolded, and those between|.3| and|.49| are underlined.

The results replicated many of the findings of the 1PL regression model, mainly that impact and uniform DIF effect size were the largest sources of influence for DIF effect size measures. The effect of impact on SIDS and SIDN grew from the previous model, but the effect on UIDS and UIDN decreased. Other signed measures (e.g., ESSD, D-max, and SA) were also influenced by impact.

Regarding uniform DIF effect size, unsigned measures (e.g., UIDS, UIDN, UA, NCDIF, RMWSD, MH Alpha) were less influenced than signed measures (e.g., SIDS, SIDN, D-Max, SA, STD, MH Delta), with the exception of Pseudo R-squared. However, it is important to note that the relationship between uniform DIF effect size and signed measures was negative, suggesting that the measures decreased for every standard deviation increase in uniform DIF effect size. Negative signs are caused by the simulation design, in which increasing uniform DIF translated to increasing the difficulty of the focal group, forcing expected scores for the reference group to be higher than those for the focal group. For most measures, a negative score indicates that the reference group is higher, so the current simulation design would produce more negative-keyed DIF effect size measures than positive-keyed ones. Therefore, as the uniform DIF effect size increases, there would be a decrease in the value of effect size measures, though the absolute value of the bias is increasing. Furthermore, the difference between the relationship of signed measures to uniform DIF effect size and that of unsigned measures to uniform DIF effect size comes down to the variability of the measures themselves. For example, the estimators for SIDS and UIDS are the same, except for the absolute value transformation in the UIDS. The variability of the SIDS cancels out due to canceling signs (i.e., positive and negative bias estimates), which delivers an unbiased estimate of uniform DIF. In contrast, the variability of the UIDS does not cancel out because the absolute values are constantly accumulating, which makes the metric less sensitive to detecting true uniform DIF. A similar relationship occurs between the other pairs of signed and unsigned measures, reducing the influence of uniform DIF on unsigned DIF effect size measures. However, the exception to that rule are MH Alpha and Pseudo R-squared, which perform strongly and use a different scaling mechanism for addressing crossing signs compared with that used in other measures (e.g., absolute values). Once again, MH Alpha, MH Delta, STD, and Pseudo R-squared did an excellent job of communicating uniform DIF effect size while ignoring bias from sample size, nonuniform DIF, and other distractor conditions.

#### Graded Response Model

The pattern of Pearson’s (Table 8) and Spearman’s (Supplemental Table S3) correlations for the GRM resembles the pattern identified in the 2PL model. The GRM model is similar to the 2PL model in its inclusion of a discrimination parameter, which allows for nonuniform DIF. Once again, the signed and unsigned measures are negatively correlated with each other in the presence of nonuniform DIF; however, the absolute value of the correlations is larger in the GRM than that in the 2PL model. For example, there is a strong correlation between SIDS and UIDS in the GRM, but that same correlation is moderate in the 2PL model. Although not as strong, a similar relationship occurs between SA and UA, increasing in strength between the 2PL model and the GRM.

**Table 8.** Pearson’s Correlation Matrix of DIF Effect Size Measures in GRM.

	A	B	C	D	E	F	G	H	I	J	K	L	M	N	O
A	–														
B	**–.78**	–													
C	**1**	**–.78**	–												
D	**–.77**	**1**	**–.77**	–											
E	.**88**	**–.63**	.**87**	**–.62**	–										
F	.**78**	**–.51**	.**79**	**–.50**	.**71**	–									
G	.**65**	–.29	.**64**	–.28	.**67**	.**54**	–								
H	**–.70**	.45	**–.70**	.44	**–.77**	**–.59**	**–.86**	–							
I	.**62**	–.43	.**63**	–.42	.**61**	.**69**	.42	–.47	–						
J	–.20	.43	–.20	.44	–.21	–.01	–.10	.18	–.08	–					
K	**–.67**	.**77**	**–.67**	.**77**	**–.51**	–.48	–.29	.39	–.39	.31	–				
L	**–.72**	.**86**	**–.72**	.**86**	**–.51**	**–.53**	–.28	.39	–.42	.34	.**83**	–			
M	.10	–.07	.10	–.07	.22	.08	.20	–.31	.03	–.05	.01	.08	–		
N	–.17	.22	–.17	.23	–.11	–.10	.03	–.01	–.10	.09	.26	.16	–.09	–	
O	**–.59**	.**59**	**–.60**	.**59**	**–.62**	–.47	–.38	.**61**	–.34	.22	.46	.**50**	–.32	.18	–

*Note*. A = SIDS, B = UIDS, C = SIDN, D = UIDN, E = ESSD, F = D-max, G = MH Alpha, H = MH Delta, I = SA, J =UA, K = CDIF, L = NCDIF, M = STD, N = RMWSD, O = Pseudo R-squared. *r* >|.5| are bolded, and|.3| < *r* >|.5| are underlined.

The difference in correlation size between the 2PL and the GRM is likely due to the differences between the item difficulty and discrimination parameters in the two models. Nonuniform DIF was added to the item discrimination parameter during the simulation, whereas uniform DIF was added to the item difficulty parameter. In the GRM, the number of difficulty parameters scales with the number of item response categories, whereas the discrimination parameter remains constant at 1. The GRM continues the same pattern of correlations as the 2PL model, except they are more exaggerated. In the 2PL model, signed and unsigned measures diverge because different information is being communicated by uniform and nonuniform DIF. In the GRM, different pieces of information are still being communicated, but it is inflated because the bias grows at different rates. Uniform DIF, while attached to the difficulty parameter, grows in response to item response thresholds, but nonuniform DIF does not grow at all, remaining constant with a single discrimination parameter. Therefore, the differences between signed and unsigned models are more exaggerated in the GRM, where there are more parameters for bias to emerge and grow, than in the 2PL model, where the number of parameters and the bias attached are stable.

There is no longer a dominant DIF effect size metric, which is correlated with most of the other measures. Instead, there are several measures that have been elevated in their relationship with other measures. For example, the expected score-based measures are now correlated with each other strongly, as well as CDIF, NCDIF, and Pseudo R-squared. The signed expected score-based measures are also correlated with the MH effect size measures. Just as in the 1PL and 2PL models, Pseudo R-squared is a strong DIF effect size metric, correlated with many signed and unsigned measures; however, CDIF and NCDIF exhibit just as many correlations with other measures and have higher correlations.

A linear model was further fitted to the GRM data predicting effect size with eight predictor variables, including the number of item response categories. Their standardized beta coefficients are presented in Table 9. The table follows similar trends as the 1PL and 2PL model, as conditions such as sample size, size of focal group, number of items, and number of DIF items have negligible influence on DIF effect size measures. Again, RMWSD is the only metric that is influenced by the number of items, which is consistent with the 1PL and 2PL models. The introduction of the number of item response categories does not meaningfully influence most of the measures either, confirming that the GRM functions similarly across simulation conditions to the 1PL and 2PL models. However, it is important to note that the number of item response categories did significantly influence UA. Although the unsigned measures generally exhibited a stronger influence from the number of categories than the signed measures, the influence received by UA was particularly strong because the metric relies on the “area under the item” response function. The “shape of the item” response function is heavily influenced by the number of response categories. Other unsigned measures such as UIDS and NCDIF do not directly involve the item response function but may engage the function in different ways, such as through expected scores. By directly engaging with the item response function, UA is more susceptible to influence from the variation in the number of item response categories. Ultimately, though, the number of categories is a negligible influence for most of the DIF effect size measures.

**Table 9.** Standardized Regression Coefficients Predicting DIF Effect Size Measures With GRM Simulation Conditions.

	*N*	*D*_*U*_	*J*	*N*_*d*_	*N*_*f*_	*I*	*D*_*NU*_	*N*_*CAT*_
SIDS	–.018	**–.682**	.024	.020	–.030	.082	.280	–.044
UIDS	.003	.**538**	–.038	–.030	.014	.049	–.063	.143
SIDN	–.019	**–.683**	.016	.018	–.031	.080	.285	–.043
UIDN	.003	.**535**	–.037	–.030	.014	.053	–.061	.149
ESSD	–.022	**–.727**	.042	.017	–.027	.115	.393	.086
D-max	.011	**–.520**	.011	.008	–.007	.015	.311	.097
MH Alpha	–.015	**–.549**	.001	.000	–.027	.**517**	.267	.031
MH Delta	.003	.**650**	.000	.000	.013	**–.579**	–.276	–.035
SA	–.003	–.406	.009	.007	.020	.026	.328	.070
UA	–.016	.270	–.026	–.020	–.012	–.004	–.052	.441
CDIF	.027	.479	.174	.179	.028	.022	–.024	.102
NCDIF	–.005	.439	–.034	–.025	.005	.016	–.011	.122
STD	.001	–.299	–.006	.020	–.006	.139	–.070	.021
RMWSD	–.059	.059	.436	–.027	–.036	.192	–.032	.153
R-squared	–.044	.**572**	.010	–.069	.276	–.062	–.118	–.062

*Note*. *N* = sample size, *D*_*U*_ = uniform DIF effect size, *J* = number of items, *N*_*d*_ = number of DIF items, *N*_*f*_ = size of focal group, *I* = size of impact, *D*_*NU*_ = nonuniform DIF effect size, and *N*_*CAT*_ is the number of item response categories. β coefficients >|.5| are bolded, and those between|.3| and|.49| are underlined.

The results for impact did not replicate the findings of the 1PL or 2PL model. The only measures that were substantially influenced by impact were MH Alpha and Delta. The MH measures were not influenced by impact in the previous models, but the MH measures in the GRM are fundamentally different from the MH measures in dichotomous IRT models. The MH measures for dichotomous models are nonparametric, using an ability level from contingency tables as a proxy for theta. In contrast, the MH measures of polytomous models rely on the cumulative odds ratio, which utilizes information from an already calculated theta. The polytomous MH measures utilize information about theta and when theta is biased due to impact: Additional bias influences the DIF effect size measures in a way that dichotomous MH measures are not influenced.

More measures were influenced by nonuniform DIF in the GRM than in the 1PL or 2PL models, but the overall influence of the condition was negligible among DIF effect size measures. All the beta coefficients were less than a .4 standard deviation increase. Again, the signed measures were observed to be more influenced by nonuniform DIF than the unsigned measures.

Naturally, the largest source of influence in the GRM comes from uniform DIF, indicating that the DIF effect size measures are mostly functioning as intended by ignoring influence from other conditions. Unlike the 1PL and 2PL models, STD is not strongly influenced by uniform DIF in the GRM, likely due to the fact that the difference statistic is built upon comparisons between dichotomous models regarding the item response function. The difference statistic does not adjust well for polytomous models, which means it is unable to capture the uniform DIF as it grows with each item response threshold. This claim is corroborated by the worsened performance of RMWSD in the GRM, which utilizes the same difference statistic as STD. Another difference is that all expected score-based measures are strongly influenced by uniform DIF, including the unsigned measures. MH Alpha, MH Delta, and Pseudo R-squared continue to be strong indicators of uniform DIF though the MH measures are shown to be biased by impact. For the GRM, it is more appropriate to describe Pseudo R-squared and the expected score-based measures as effective communicators of uniform DIF effect size with limited influence from other sources of bias.

## Discussion

### Findings

Three separate simulation studies were conducted for the 1PL, 2PL, and GRM models, respectively, to understand the consensus among DIF effect size measures. The primary aim was to understand which DIF effect size measures agreed with each other, particularly regarding the size of uniform DIF. Nested within that aim was a secondary goal to identify which measures were able to communicate the most unique information about uniform DIF, containing as little overlap as possible with other DIF effect size measures. Finally, it is important to identify which DIF effect size measures are least influenced by data characteristics which may constitute bias. Selectively reporting DIF effect size measures that meet these three criteria will reduce the total number of effect sizes to report, while also preserving measures that are the most valid indicators of true DIF effect size in the population, improving interpretability and accuracy.

#### 1PL Model

Given the lack of a discrimination parameter, uniform DIF was the only factor that influences DIF effect size measures. For the 1PL model, five clusters of measures emerged among the Pearson’s correlations. First, expected score-based measures, Raju’s area methods, STD, and NCDIF tended to be strongly correlated with each other, forming a single cluster. It is important to note that this cluster includes both signed and unsigned measures. Next, the MH measures were strongly correlated with each other and distinct from the other parametric methods. Next, CDIF was the only compensatory DIF metric, which had slightly lower correlations than the first cluster and was also distinctly not correlated with the MH measures, suggesting that it formed a cluster by itself. Similarly, RMWSD was not correlated with anything strongly, so it also formed a cluster by itself. Finally, Pseudo R-squared was correlated with most of the other DIF effect size measures, yet the correlations were not quite as strong as the first cluster, suggesting that it is a unique metric that is also highly related to the other measures. Measures that share the same clusters are highly similar to each other and communicate the same information about uniform DIF effect size, meaning that reporting one in a study would be sufficient to capture a given effect.

The linear regression results provide more insights as to which metric to report within each cluster. The first cluster is the hardest to provide a single recommendation for in the 1PL model because each metric has distinct advantages and disadvantages. First, whether to use the sample-based measures or the normal distribution-based measures is dependent on available data and whether it is believed that the estimated theta in the sample follows a normal distribution. If theta is normal, the two types of measures communicate the same information perfectly in a 1PL model, and it does not matter which metric is selected. However, if theta is not normal, the normal distribution–based measures have a clear advantage. Of course, there is also the dilemma of choosing between a signed and an unsigned metric, which depends on whether the sign of DIF is theoretically important to the researcher. If directionality is not important, UIDS and UIDN are slightly less influenced by sample size, size of focal group, and number of items, but the effect is negligible regardless. Finally, SA and UA have the highest influence from impact within the cluster, but they are the most influenced by uniform DIF effect size, indicating that they retain the effects of DIF in the population the strongest. Ultimately, SA makes the strongest case for being the single metric to represent this cluster, particularly if directionality of DIF is important; however, researchers should select SIDS instead if impact is suspected within the population.

For the cluster containing MH measures, the decision is much easier. MH Delta communicates uniform DIF effect size more strongly than MH Alpha and is also less susceptible to bias from sample size, size of focal group, number of items, number of DIF items, and impact. MH Delta is also more interpretable, possessing empirical cutoff indices that are widely used within the field. Although the present study did establish indices for MH Alpha, the indices for MH Delta have a much longer history of validation and should be prioritized.

There is little reason to use the single metric clusters containing CDIF and RMWSD, both of which are metrics least influenced by uniform DIF effect size. Each metric does a poor job of communicating the DIF effect in the population, and they are also highly biased by other factors, such as impact and the number of items. The only time using CDIF is appropriate is if there is a theoretical justification for examining the effect of compensatory DIF in the sample, but there is no justification for using RMWSD.

The final cluster, containing Pseudo R-squared, is the best of all worlds because it is highly influenced by uniform DIF effect size and similar to most of the other DIF effect size measures, yet distinct enough to offer unique information. However, one disadvantage is that it is influenced significantly by the size of the focal group, since all Pseudo R-squared measures are susceptible to class imbalance (Smith & McKenna, 2013). Ultimately, if a researcher had to select only one DIF effect size metric for the 1PL model, MH Delta is the best metric to report, but the strongest strategy is to report multiple measures including MH Delta, SA, and Pseudo R-squared.

#### 2PL Model

The introduction of the discrimination parameter and nonuniform DIF drastically changed the structure of the correlation matrices. Now, six clusters emerge among the correlations: signed measures, unsigned measures, MH measures, CDIF, RMWSD, and Pseudo R-squared. Just like in the 1PL model, it is easy to discard the clusters containing CDIF and RMWSD given their poor performance and niche use cases. The advantages of MH Delta over MH Alpha are also consistent with those in the 1PL model, providing an easy decision to report MH Delta among a list of DIF effect size measures. Pseudo R-squared becomes increasingly more important because despite the differences between signed and unsigned measures, it shares information with all of them and becomes a bridge between conflicting outcomes. Either MH Delta or Pseudo R-squared makes a compelling case for being single reportable measures in the 2PL model.

Again, the signed and unsigned metric clusters are more difficult to disentangle given an assortment of advantages and disadvantages. Regarding the signed measures, SA is no longer the metric most influenced by uniform DIF effect size. The effect has been much attenuated in the 2PL model. In addition, SA is highly susceptible to extreme values and outliers in the 2PL model, which has hazardous implications for empirical studies. All the expected score-based measures are also highly susceptible to influence from impact and may not be appropriate in some DIF applications. STD is the best choice for the 2PL model because it has the strongest influence from uniform DIF effect size and the lowest influence from most of other simulation conditions, the exception being nonuniform DIF effect size. An important consideration for the choice of signed or unsigned metric is whether the unsigned metric offers new information that the signed metric does not. If an unsigned metric is valuable, such as in the case of crossing signs, UIDS and UIDN are the most influenced by uniform DIF effect size, with little influence from all other conditions. That said, Pseudo R-squared is also unsigned and shares many of the same advantages, in addition to other distinct advantages; therefore, it may be more appropriate to report Pseudo R-squared in place of a specific unsigned metric of this cluster. Overall, the combined reporting of STD, MH Delta, and Pseudo R-squared provides the most unique information about uniform DIF effect size in a 2PL model.

#### Graded Response Model

The pattern identified in the 2PL model was similarly replicated in the GRM, although a greater number of clusters were observed. Nine clusters were observed, as many measures diverged from their previous clusters and formed their own associations in the GRM. Measures that formed their own clusters included SA, UA, CDIF, STD, RMWSD, and Pseudo R-squared. Again, there is limited reason to report CDIF and RMWSD, except in niche investigations. Furthermore, SA, UA, and STD are uninfluenced by uniform DIF effect size and should not be considered as a valid indicator of the effect in the population. Pseudo R-squared continues to be a valid single reportable metric, with the same advantages and disadvantages as in the 1PL and 2PL models.

The MH measures formed their own cluster once more in the GRM. The relationship between uniform DIF effect size and the MH measures is lower in the GRM than in the other models, but it still provides valid and unique information about DIF in the population. However, a new disadvantage of the cumulative odds ratio approach with the MH measures is that it is highly susceptible to bias from impact. Furthermore, the advantage of using empirically developed cutoff indices for absolute interpretation is also reduced. That said, MH Delta is still a valuable metric to report when impact is not suspected within the data.

There are many options available for signed and unsigned measures. ESSD is most strongly influenced by uniform DIF effect size, but it is also influenced significantly by nonuniform DIF. SIDS or SIDN, which have less influence from nonuniform DIF, may be a more appropriate choice to represent signed measures. SIDS and SIDN also have limited influence from all other conditions, making them an overall less-biased choice. Again, the choice of SIDS or SIDN depends on the assumption of normality within the sample; otherwise, the measures are equivalent. That said, it is generally safer to use SIDN than SIDS. The final recommendations for the GRM are to report SIDN, MH Delta, and Pseudo R-squared.

### Summary of Findings

Although all 15 DIF effect size measures tend to have consensus with each other, a select few measures communicate unique information about uniform DIF effect size, which should be prioritized in reporting DIF results. For each model, we recommend that researchers report one signed, one unsigned, and one nonparametric DIF effect size measure. The signed measure shows the directionality of DIF, the unsigned measure shows the magnitude, and the nonparametric measure may indicate how DIF would appear differently if a different model (e.g., 1PL, 2PL, GRM) was specified, which together represent the totality of what is known about the DIF effect. Regardless of model, MH Delta and Pseudo R-squared are always valuable to report as nonparametric and unsigned DIF effect size measures. However, the proper signed metric to report varies by model. SA is best for the 1PL model, STD for the 2PL model, and SIDN (or SIDS) for the GRM.

### Limitations and Future Directions

Although the present study advanced current understanding of the consensus among DIF effect size measures and improved the interpretability of measures, there are multiple limitations that can be addressed in the future. Naturally, the present study is a simulation study. Monte Carlo simulation studies are effective at investigating the properties of statistical methods and measures within a known population, but there is no way to know how closely the simulated data resembles data in the real world. The agreements of the effect size measures can be investigated through real data sets.

Although the list of DIF effect size measures investigated in the current study was comprehensive, there are a variety of other measures that were not considered, such as the many other types of Pseudo R-squared besides McFadden’s Pseudo R-squared. DIF effect size measures that were too similar to other measures were excluded from the study to maximize the amount of unique information learned about DIF effect size measures. Still, future studies may consider investigating measures not discussed in the present study to develop a more comprehensive understanding of the consensus among DIF effect size measures. The present study also only examined the relationships among measures across simulation conditions. It would be valuable to further investigate these relationships within simulation conditions to understand the situations in which consensus changes in response to a covariate.

There are also many criteria which one may use to declare what makes a measure a “good” effect size measure. The present study employed three criteria: unique information, strong influence from true uniform DIF effect size, and lack of bias (e.g., impact). However, others may assert that other criteria are more important. It is important that the conversation surrounding DIF effect size and their utility continues to develop so that researchers in the social and behavioral sciences are given the best tools available to make decisions about measurement bias.

## Supplemental Material

sj-docx-1-epm-10.1177_00131644261458305 – Supplemental material for Consensus Among Differential Item Functioning Effect Size Measures: A Simplified Approach to Reporting Effect Size Supplemental material, sj-docx-1-epm-10.1177_00131644261458305 for Consensus Among Differential Item Functioning Effect Size Measures: A Simplified Approach to Reporting Effect Size by Austin Wyman and Zhiyong Zhang in Educational and Psychological Measurement
