# Supplementary material for: Consensus Among Differential Item Functioning Effect Size Measures: A Simplified Approach to Reporting Effect Size
Source: Educ Psychol Meas. 2026 Jul 15:00131644261458305. Online ahead of print. doi: 10.1177/00131644261458305 (PMC13375857; doi:10.1177/00131644261458305)
Supplement: sj-docx-1-epm-10.1177_00131644261458305 – Supplemental material for Consensus Among Differential Item Functioning Effect Size Measures: A Simplified Approach to Reporting Effect Size [file sj-docx-1-epm-10.1177_00131644261458305.docx]

**Supplementary Material**

**Literature Review of DIF Effect Size Measures**

**Mantel-Haenszel Measures.** In the case of dichotomous items, the Mantel-Haenszel (MH) test constructs a J – 1 number of 2 x 2 contingency tables of group versus outcomes, in which J is the total number of items (Mantel & Haenszel, 1959). Each contingency table corresponds to a different total score, as responses are grouped by matched total score, which is a proxy for ability in nonparametric methods. A chi-square test is conducted to evaluate whether the proportion of correct responses in the reference group is different from its expectation across the different score levels. The null hypothesis for the MH test is that no relationship exists between group membership and the item response, controlling for total score (Li, 2015). The alternative hypothesis is that a relationship does exist, but it provides no information regarding which group the relationship benefits. Hence, effect size measures are necessary to determine the true nature of the relationship.

An odds-ratio can be computed from the 2 x 2 contingency table, where a value greater than 1 indicates that the reference group is more likely to receive a correct response, less than 1 indicates the focal group is more likely, and exactly 1 indicates that the groups are equally likely. Odds-ratios are obtained within each score group, but may be aggregated together via a common odds-ratio. MH Alpha is the common odds-ratio of the contingency tables, which describes the likelihood of higher scores being observed in the reference group versus focal group across all score levels (Holland & Thayer, 1986). It can be expressed in the following equation,

|  | $\hat{\alpha}_{MH}= \frac{\sum_{j} {A_{j}D_{j}}/{N_{j}}}{\sum_{j} {B_{j}C_{j}}/{N_{j}}} ,$ | (2) |
| --- | --- | --- |

where $A_{j}$ is the number of correct responses of the reference group in the j^th^ score level, $D_{j}$ is the number of incorrect responses in the focal group, $B_{j}$ is the number of correct responses in the focal group, and $C_{j}$ is the number of incorrect responses in the reference group. MH Alpha scores can range from 0 to infinity. Values less than 1 (i.e., closer to 0) indicate that the DIF effect favors the focal group, whereas values greater than 1 (i.e., closer to infinity) indicate that the effect favors the reference group. Alpha values equal to 1 indicate that no DIF is present.

Because it is difficult to compare MH Alpha that is above or below 1, a “delta” rescaling of MH-alpha was recommended by Holland and Thayer (1986), which rescales the index using the natural log and a constant,

|  | $\hat{\Delta}_{MH}=-\frac{4}{1.7} ln\left( \hat{\alpha}_{MH} \right)=-2.35 ln\left( \hat{\alpha}_{MH} \right) .$ | (3) |
| --- | --- | --- |

MH Delta has increased interpretability, as the index is now distributed symmetrically with a value of 0 indicating no DIF. Positive values of MH Delta indicate that the focal group has higher odds, meaning the item is less difficult for the focal group. Negative values indicate an item is more difficult for the focal group.

The MH test and the delta effect size are often used together. The Educational Testing Service (ETS) classifies DIF into three categories: A items, B items, and C items (Zwick, 2012; Zwick & Ercikan, 1989). Class “A” items represent negligible DIF and are classified when either the chi-square test is not significant or the absolute value of MH-delta is less than 1. “C” items represent severe DIF and are classified when both the chi-square test is statistically significant and the absolute value of MH Delta is greater than 1.5. Finally, “B” items represent moderate DIF and are classified as any item that doesn’t meet the criteria for either A or B. The ETS cutoffs rely on the MH chi-square test and MH Delta, but since the two statistics tend to align with one another, it is also common for DIF to be classified into these categories based on the effect size cutoffs alone (Magis et al., 2015).

The original MH approach must be altered in the case of polytomous items, expanding the 2 x 2 contingency table to 2 x K dimensions, where K is the number of response categories for the item (Wang & Su, 2004). A new chi-square statistic is also constructed for the “Mantel” approach, which weights the proportion of item responses by their response value. Effects size measures may still be computed. Instead of a common odds-ratio, Liu & Agresti’s (1996) cumulative odds-ratio is computed, which estimates the difference in response pattern by group across score levels, while weighing the relative values of the ordinal responses. Although the properties of a delta equivalent have not be discussed in the literature, Penfield & Algina (2003) suggested that the similar log scale can be applied to the cumulative odds-ratio to improve its interpretability.

**Item Discrepancy Indices.** Two other nonparametric effect size measures that utilize contingency tables are known as the “Item Discrepancy Indices” (Dorans & Kulick, 1983). The first and simplest index is the P-difference index, *D_s_*, which is the difference in conditional probability between the reference and focal groups evaluated for every score group, *s*. *D_s_* is unidirectional, with positive values indicating that the item bias favors the focal group and negative values indicating bias favors the reference group. Sometimes the squared P-difference, $D_{s}^{2}$, is used instead, which measures the absolute strength of difference and ignores the interpretation of DIF direction. Both variations of the P-difference index are limited by the lack of aggregation across score levels, which makes the difference index more appropriately a measure of impact, not DIF. However, *D_s_* can be transformed into a more suitable DIF index through the insertion of weights, such as in the standardized P-difference (STD) and the Root Mean Weighted Square Difference (RMWSD). STD stands as the effect size corresponding to the SIBTEST and can be expressed as the following equation (Dorans & Kulick, 1983),

|  | $STD={\sum_{s} k_{s}D_{s}}/{\sum_{s} k_{s} =}{\sum_{s} k_{s}\left( P_{Fs}-P_{Rs} \right)}/{\sum_{s} k_{s}},$ | (4) |
| --- | --- | --- |

where $k_{s}$ are the weights applied for each score level. Dorans & Kulick (1986) propose multiple values that can be used to standardize the *D_s_*, though it is recommended that *k_s_* represent the number of respondents in the focal group within a score group, *N_Fs_*, so that the standardization places the greatest weight on the focal group. STD can be interpreted similarly as *D_s_*, where positive values indicate that DIF benefits the focal group and negative values indicate DIF benefits the reference group. The directionality of it is easier to interpret, but may be biased by cancellation effects, particularly at the score level. The RMWSD can be used alternatively to obtain an absolute difference, given by (Dorans & Kulick, 1983),

|  | $RMWSD = \sqrt{{\sum_{s} k_{s}D_{s}^{2}}/{\sum_{s} k_{s}}} = \sqrt{{\sum_{s} k_{s}\left( P_{Fs}-P_{Rs} \right)^{2}}/{\sum_{s} k_{s}}} .$ | (5) |
| --- | --- | --- |

Both STD and RMWSD are referred to cutoffs that have been determined empirically from large sample educational assessments (Dorans & Kulick, 1983; Dorans & Kulick, 1986). For STD, absolute values greater than or equal to 0.05 are flagged for DIF, with values greater than 0.10 being flagged as particularly severe. For RMWSD, item with values greater than 0.08 are flagged for DIF, with additional, higher standard cutoffs at 0.16 and 0.24. Simulation and empirical studies suggest that the STD and MH approaches agree in DIF detection with one another (Dorans & Holland, 1993), but the STD approach has higher power than MH-delta but much larger Type I error rates (Fidalgo et al., 2004). However, the method was developed entirely for dichotomous item response data and does not currently support polytomous items, limiting its overall utility compared to approaches like the Mantel approach or GMH test. That said, since STD is an effect size for the SIBTEST, the coefficient for the Poly-SIBTEST, corresponding to polytomous items, may be used a stand-in for a STD-equivalent effect size for polytomous items.

**Expected Score-based Measures.** The most direct effect size that can be obtained from an IRT model is the expected score (ES), which describes the model-implied score a respondent would receive according to their estimated ability level and the conditional probability of observing an item response given ability. The ES is a fundamental feature of IRT models and can be obtained from dichotomous, polytomous, and even nonparametric IRFs, although its usage is preferred for parametric DIF detection rather than nonparametric. The ES for a given participant, *s*, and item, *j*, is given by the following equation, (Meade, 2010),

|  | $ES_{\mathrm{ij}}=\sum_{k=1}^{m} P_{jk}\left( \hat{\theta}_{i} \right)X_{jk},$ | (6) |
| --- | --- | --- |

where *m* is the total number of response categories, $P_{jk}\left( \hat{\theta}_{i} \right)$ is the IRF (the conditional probability of observing the k^th^ response on the j^th^ item) according to the item parameters estimated from the selected IRT model, and $X_{jk}$ is the value of the response option. The formula above is specified for polytomous models, but generalizes well to dichotomous models, given that $X_{jk}$ is confined to 0 or 1 for binary items, which reduces the equation to the probability of obtaining a response of X = 1. The ES corresponding to the reference and focal group can be obtained by substituting the full sample item parameters in the IRF with item parameters from the reference and focal group respectively. Because the group-level ES is such a fundamental parameter for IRT models, it serves as the basis of multiple effect size measures for DIF, including the Signed Item Difference in the Sample (SIDS), the Unsigned Item Difference in the Sample (UIDS), the Maximum Difference in the Sample (D-Max), and the Expected Score Standardized Difference (ESSD).

The SIDS and UIDS are signed and unsigned expressions of the average difference in the group-level ES across the sample. The SIDS is given by the following equation (Meade, 2010),

|  | $SIDS_{j}= {\sum_{i=1}^{N} \left[ {ES}_{\left( ij \vert\hat{\theta},\gamma_{F} \right)} - {ES}_{\left( ij \vert\hat{\theta},\gamma_{R} \right)} \right]}/N ,$ | (7) |
| --- | --- | --- |

where ${ES}_{\left( ij | \hat{\theta},\gamma_{F} \right)}$ and ${ES}_{\left( ij | \hat{\theta},\gamma_{R} \right)}$ are the group-level ES for the focal and reference group respectively. The SIDS is interpreted directionally, with positive values indicating greater DIF benefitting the focal group, negative values indicating greater DIF benefitting the reference group, and values approximating 0 indicating no DIF is present. However, as a signed index, the SIDS is vulnerable to DIF cancellation effects. The effect size will shrink toward 0 as it sums across all respondents’ group differences, if there is a mixture of positive and negative values. The UIDS may be used as a solution, which is given by a matching expression to the SIDS (Meade, 2010),

|  | $UIDS_{j}={\sum_{i=1}^{N} \left\vert{ES}_{\left( ij \vert\hat{\theta},\gamma_{F} \right)} - {ES}_{\left( ij \vert\hat{\theta},\gamma_{R} \right)} \right\vert}/N .$ | (8) |
| --- | --- | --- |

The SIDS and UIDS produce equivalent results in cases where there is uniform DIF (except that the signs may be swapped). However, the SIDS and UIDS may differ greatly in the presence of nonuniform DIF. Given that the UIDS communicates unique information about DIF cancellation, which the SIDS does not, Meade (2010) recommends that the two effect size measures are used together.

Averaging difference scores across the sample is vulnerable to shrinkage from aggregation and cancellation effects, potentially biasing the SIDS and UIDS effect size estimates. Aggregation issues can be circumvented by taking the maximum in the sample instead of the mean. The D-Max index describes the maximum difference in ES between the reference and focal group in the sample. Although D-Max avoids bias via aggregation and cancellation effects, it is vulnerable to bias from outliers, such that extreme differences in ESs may not be representative of the IRT model overall. Thus, there is a tradeoff between SIDS, UIDS, and D-Max, often resulting in researchers reporting them together.

Another limitation of the SIDS, UIDS, and D-Max is that they are based on observed DIF in the sample, which may not generalize to the population. In particular, there is concern that the distribution of theta in the sample does not resemble the assumed normal distribution in the population. Normal distribution-based extensions of the SIDS and UIDS are available, producing SIDN and UIDN, respectively. The approach generates a normal distribution of theta values, either through integration or more commonly, Gaussian–Hermite quadrature nodes (Meade, 2010). Weights for each theta are obtained from density weights corresponding to the probability density function of the normal distribution. The weights modify the difference in ES from the original UIDS and SIDS equations, resulting in the following modified equations (Meade, 2010),

|  | $SIDN_{j}=\sum_{\theta} \left[ ES_{\theta,\gamma F}-ES_{\theta,\gamma R} \right]w_{\theta} ,$ | (9) |
| --- | --- | --- |
|  | $UIDN_{j}=\sum_{\theta} \left[ \left\vert ES_{\theta,\gamma F}-ES_{\theta,\gamma R} \right\vert\right]w_{\theta},$ | (10) |

where $w_{\theta}$ is the probability density weights for each theta. The interpretation of the SIDN and UIDN matches the interpretation of the SIDS and UIDS, with respect to magnitude and directionality. The primary advantage of the normal distribution-based effect size measures is that offer more robust estimates compared to their sample counterparts, yet SIDN and UIDN still rely on item parameters estimated by the sample, which provide opportunities for bias to persist regardless.

The final ES-based metric is the ESSD (Meade, 2010), which has been described as the ES version of Cohen’s *d* (Cohen, 1988). ESSD is normed statistic that describes the mean difference in ES between the reference and focal group in units of pooled standard deviation. The pooled standard deviation for an item is given by the following equation (Meade, 2010),

|  | $SD_{Pooled}=\sqrt{\frac{\left( N_{F}-1 \right)\sigma_{ES\left( j \vert\gamma F \right)}^{2}+\left( N_{F}-1 \right)\sigma_{ES\left( j \vert\gamma R \right)}^{2}}{2*N_{F}-2}} ,$ | (11) |
| --- | --- | --- |

where $N_{F}$ is the sample size of the focal group, $\sigma_{ES\left( j | \gamma F \right)}^{2}$ is the variance in ES for the j^th^ item with the focal group item parameters, and $\sigma_{ES\left( j | \gamma R \right)}^{2}$ is the variance in ES for the reference group. Therefore, the ESSD can be obtained through the following equation (Meade, 2010),

|  | $ESSD = \frac{\bar{ES}_{\left( \gamma F \right)}-\bar{ES}_{\left( \gamma R \right)}}{SD_{Pooled}} .$ | (12) |
| --- | --- | --- |

ESSD values can easily be interpreted with respect to magnitude and directionality. Positive values indicate the DIF effect favors the focal group, whereas negative values favor the reference group. ESSD units are expressed as standard deviation differences between the reference and focal group, which allows for their interpretation of their magnitude according to Cohen’s *d* guidelines. Absolute values of ESSD between 0.2 and 0.5 are considered small, between 0.5 and 0.8 are considered moderate, and values above 0.8 are considered strong. Cutoff values are a particular strength of the ESSD, as it improves interpretability; however, no empirical work has established similar cutoffs for the other ES-based measures.

**Pseudo R^2^.** Multiple versions of Pseudo R^2^ that have been proposed for DIF over time, including measures developed by McFadden (1974), Cox and Snell (1989), Nagelkerke (1991), and Tjur (2009). McFadden’s Pseudo R^2^ observes the ratio in log likelihood between the full model and null model, which is given by the following equation (McFadden, 1974),

|  | $R_{MF}^{2}=1-\frac{LL_{M}}{LL_{0}} ,$ | (13) |
| --- | --- | --- |

where $LL_{M}$ is the log likelihood of the full model and $LL_{0}$ is the log likelihood of the null model, which contains no predictors. McFadden’s Pseudo R^2^ has a minimum of 0 and maximum of 1.

There have been multiple proposed approaches to interpreting Pseudo R^2^. Zumbo (1999) recommended using Cohen’s R^2^ guidelines for effect size (1992), which argue that values below 0.13 are negligible in effect size, values between 0.13 and 0.26 are moderate, and values above 0.26 are large. Jodoin and Gierl (2001) proposed the following guidelines for Pseudo R^2^: values of ΔR^2^ less than 0.035 are negligible (“A” items), values greater than or equal to 0.070 are large (“C” items), and all other values are moderate (“B” items). However, evidence suggests that the current cutoff approaches are not sensitive to empirical DIF effect sizes, with the current indices failing to classify large and moderate-sized DIF correctly (Hidalgo & Lopez-Pina, 2004; French & Maller, 2007). Although the Pseudo R^2^ approach lacks the power of MH Delta for uniform DIF, it is superior in its ability to detect multiple types of DIF, particularly nonuniform DIF. Pseudo R^2^ has its greatest utility when it is used in combination with a corresponding statistical test (e.g., LRT), resulting in Type I error rates close to 0 (Gomez-Benito et al., 2009).

**Effect Size Extensions of Statistical Tests.** There is also a class of DIF effect size measures that are nested within DIF statistical tests. Raju (1990) proposed two measures: the signed area (SA) and the unsigned area (UA) to operationalize the difference. The SA integrates the IRF for the reference and focal group separately and describes the difference unidirectionally, which is given by the following equation,

|  | $SA = \int_{-\infty}^{\infty} \left( \hat{F}_{R}-\hat{F}_{F} \right)d\theta,$ | (14) |
| --- | --- | --- |

where $\hat{F}_{R}$ and $\hat{F}_{F}$ are the IRFs for the reference group and focal group respectively. The SA is not intended to be applied to any specific IRT model in particular, such as in Lord’s test. Any IRF can be inserted into the SA function. UA integrates the IRFs similarly, except that the absolute value of the difference between areas is used rather than the signed difference. The UA is given by the following equation,

|  | $UA = \int_{-\infty}^{\infty} \left\vert\hat{F}_{R}-\hat{F}_{F} \right\vert d\theta.$ | (15) |
| --- | --- | --- |

The overall process of obtaining the SA and UA is consistent across different types of IRT models, but integral looks differently according to which IRT model is specified and whether the group discrimination parameters are equal to another (Raju, 1990). As an effect size, positive SA values indicate that the reference group has a higher conditional probability of receiving a correct response than the focal groups, negative values indicate the opposite, and values close to 0 indicates that there is little difference in probability. The UA cannot be interpreted directionally, but greater UA values indicate greater absolute DIF between the groups and smaller values indicate less DIF.

Another example is found with the Differential Functioning of Items and Tests (DFIT) procedure (Raju et al., 1995). The DFIT procedure is similar to the area methods in that it evaluates the difference in IRFs between the reference group and focal group for each respondent and item, *d*, except that the difference here is weighted by *D*, or the sum of *d* values across the sample. Hence, a measure of compensatory DIF (CDIF) is produced by the following equation,

|  | $CDIF_{j}=E\left( d_{j}D \right)=Cov\left( d_{i},D \right)+\mu_{d_{j}}\mu_{D} .$ | (16) |
| --- | --- | --- |

The term compensatory here means that the measure is compensating for bias and attempting to spread the bias across multiple locations. The dispersion can occur in multiple ways, but here the DIF effect is dispersed across all items due to the influence of *D*. The compensatory nature of the measure can act as a measure of relative importance as well, given that it models the covariance between the item bias and scale bias. Higher CDIF values indicate not only that an item demonstrates DIF, but that eliminating the item would improve differential test functioning for the remainder of the scale.

The DFIT procedure may produce a noncompensatory measure DIF as well, known as NCDIF, which isolates just the effect of item-level bias, without controlling for the effect of test-level bias. NCDIF is produced by removing the influence of *D* from the CDIF equation,

|  | $NCDIF_{j}=\sigma_{d_{j}}^{2}+\mu_{d_{j}} .$ | (17) |
| --- | --- | --- |

Among the two, NCDIF is more often used as an effect size. Wright and Oshima (2015) recommend the following cutoff ranges for NCDIF: values less than 0.003 are negligible, values between 0.003 and 0.008 are moderate, and values greater than 0.008 are large. However, the size of NCDIF should be interpreted in the context of the size of the corresponding model’s item parameters, making the effect size metric more challenging to interpret than MH-delta or Pseudo R^2^, which have stable effect size guidelines.

**Supplementary Tables for Results Section**

| Table S1. Spearman’s correlation matrix of DIF effect size measures in 1PL model. | | | | | | | | | | | | | | | | | | | | | | | | | | | | | |  | |
| --- | --- | --- | --- | --- | --- | --- | --- | --- | --- | --- | --- | --- | --- | --- | --- | --- | --- | --- | --- | --- | --- | --- | --- | --- | --- | --- | --- | --- | --- | --- | --- |
|  | A | | B | | C | | D | | E | | F | | G | | H | | I | | J | | K | | L | | M | | N | | O | |  |
| A | - | |  | |  | |  | |  | |  | |  | |  | |  | |  | |  | |  | |  | |  | |  | |  |
| B | **1** | | - | |  | |  | |  | |  | |  | |  | |  | |  | |  | |  | |  | |  | |  | |  |
| C | **1** | | **1** | | - | |  | |  | |  | |  | |  | |  | |  | |  | |  | |  | |  | |  | |  |
| D | **1** | | **1** | | **1** | | - | |  | |  | |  | |  | |  | |  | |  | |  | |  | |  | |  | |  |
| E | **.91** | | **.90** | | **.91** | | **.91** | | - | |  | |  | |  | |  | |  | |  | |  | |  | |  | |  | |  |
| F | **.85** | | **.85** | | **.87** | | **.87** | | **.92** | | - | |  | |  | |  | |  | |  | |  | |  | |  | |  | |  |
| G | **.69** | | **.69** | | **.70** | | **.70** | | **.78** | | **.83** | | - | |  | |  | |  | |  | |  | |  | |  | |  | |  |
| H | **-.69** | | **-.69** | | **-.70** | | **-.70** | | **-.78** | | **-.83** | | **-1** | | - | |  | |  | |  | |  | |  | |  | |  | |  |
| I | **.82** | | **.82** | | **.84** | | **.84** | | **.92** | | **.99** | | **.84** | | **-.84** | | - | |  | |  | |  | |  | |  | |  | |  |
| J | **.82** | | **.82** | | **.84** | | **.84** | | **.92** | | **.99** | | **.84** | | **-.84** | | 1 | | - | |  | |  | |  | |  | |  | |  |
| K | **.77** | | **.77** | | **.78** | | **.78** | | **.73** | | **.77** | | .38 | | -.38 | | **.75** | | **.75** | | - | |  | |  | |  | |  | |  |
| L | **.99** | | **.99** | | **.99** | | **.99** | | **.93** | | **.90** | | **.74** | | **-.74** | | **.88** | | **.88** | | **.79** | | - | |  | |  | |  | |  |
| M | **.81** | | **.80** | | **.80** | | **.80** | | **.73** | | **.69** | | **.85** | | **-.85** | | **.66** | | **.66** | | .37 | | **.80** | | - | |  | |  | |  |
| N | .45 | | .45 | | .46 | | .46 | | .40 | | .49 | | .47 | | -.47 | | .47 | | .47 | | .46 | | .47 | | .48 | | - | |  | |  |
| O | **.67** | | **.67** | | **.68** | | **.68** | | **.70** | | **.72** | | **.87** | | **-.87** | | **.71** | | **.71** | | .33 | | **.70** | | **.84** | | .48 | | - | |  |
| *Note*. A = SIDS, B = UIDS, C = SIDN, D = UIDN, E = ESSD, F = D-max, G = MH Alpha, H = MH Delta, I = SA, J =UA, K = CDIF, L = NCDIF, M = STD, N = RMWSD, O = Pseudo R-squared. *r* > \|.5\| are bolded, \|.3\| < *r* > \|.5\| are underlined. | | | | | | | | | | | | | | | | | | | | | | | | | | | | | |  | |
| Table S2. Spearman’s correlation matrix of DIF effect size measures in 2PL model. | | | | | | | | | | | | | | | | | | | | | | | | | | | | | | | |
|  | | A | | B | C | D | | E | | F | | G | | H | | I | | J | | K | | L | | M | | N | | O | | |  |
| A | | - | |  |  |  | |  | |  | |  | |  | |  | |  | |  | |  | |  | |  | |  | | |  |
| B | | -.38 | | - |  |  | |  | |  | |  | |  | |  | |  | |  | |  | |  | |  | |  | | |  |
| C | | **1** | | -.38 | - |  | |  | |  | |  | |  | |  | |  | |  | |  | |  | |  | |  | | |  |
| D | | -.38 | | **1** | -.38 | - | |  | |  | |  | |  | |  | |  | |  | |  | |  | |  | |  | | |  |
| E | | **.97** | | -.34 | **.97** | -.35 | | - | |  | |  | |  | |  | |  | |  | |  | |  | |  | |  | | |  |
| F | | **.86** | | -.33 | **.87** | -.34 | | **.83** | | - | |  | |  | |  | |  | |  | |  | |  | |  | |  | | |  |
| G | | **.78** | | -.26 | **.79** | -.26 | | **.77** | | **.73** | | - | |  | |  | |  | |  | |  | |  | |  | |  | | |  |
| H | | **-.78** | | .26 | **-.79** | .26 | | **-.77** | | **-.73** | | **-1** | | - | |  | |  | |  | |  | |  | |  | |  | | |  |
| I | | **.87** | | -.31 | **.88** | -.32 | | **.87** | | **.92** | | **.70** | | **-.70** | | - | |  | |  | |  | |  | |  | |  | | |  |
| J | | -.25 | | **.67** | -.25 | **.69** | | -.27 | | -.28 | | -.09 | | .09 | | -.30 | | - | |  | |  | |  | |  | |  | | |  |
| K | | .35 | | .38 | .35 | .38 | | .35 | | .26 | | .39 | | -.39 | | .29 | | .29 | | - | |  | |  | |  | |  | | |  |
| L | | -.37 | | **.96** | -.37 | **.97** | | -.33 | | -.37 | | -.28 | | .28 | | -.34 | | **.72** | | .39 | | - | |  | |  | |  | | |  |
| M | | **.83** | | -.33 | **.83** | -.33 | | **.80** | | **.72** | | **.93** | | **-.93** | | **.73** | | -.13 | | .40 | | -.31 | | - | |  | |  | | |  |
| N | | -.31 | | .26 | -.32 | .27 | | -.26 | | -.36 | | -.35 | | .35 | | -.36 | | .24 | | -.10 | | .25 | | -.44 | | - | |  | | |  |
| O | | **-.65** | | .42 | **-.66** | .43 | | **-.64** | | **-.60** | | **-.83** | | **.83** | | **-.59** | | .29 | | -.30 | | .45 | | **-.77** | | .39 | | - | | |  |
| *Note*. A = SIDS, B = UIDS, C = SIDN, D = UIDN, E = ESSD, F = D-max, G = MH Alpha, H = MH Delta, I = SA, J =UA, K = CDIF, L = NCDIF, M = STD, N = RMWSD, O = Pseudo R-squared. *r* > \|.5\| are bolded, \|.3\| < *r* > \|.5\| are underlined. | | | | | | | | | | | | | | | | | | | | | | | | | | | | | | | |

| Table S3. Spearman’s correlation matrix of DIF effect size measures in GRM. | | | | | | | | | | | | | | |  | |
| --- | --- | --- | --- | --- | --- | --- | --- | --- | --- | --- | --- | --- | --- | --- | --- | --- |
|  | A | B | C | D | E | F | G | H | I | J | K | L | M | N | | O |
| A | - |  |  |  |  |  |  |  |  |  |  |  |  |  | |  |
| B | **-.74** | - |  |  |  |  |  |  |  |  |  |  |  |  | |  |
| C | **1** | **-.73** | - |  |  |  |  |  |  |  |  |  |  |  | |  |
| D | **-.73** | **1** | **-.72** | - |  |  |  |  |  |  |  |  |  |  | |  |
| E | **.93** | **-.63** | **.93** | **-.62** | - |  |  |  |  |  |  |  |  |  | |  |
| F | **.83** | **-.61** | **.84** | **-.60** | **.77** | - |  |  |  |  |  |  |  |  | |  |
| G | **.73** | -.45 | **.73** | -.44 | **.80** | **.61** | - |  |  |  |  |  |  |  | |  |
| H | **-.73** | .45 | **-.73** | .44 | **-.80** | **-.61** | **-1** | - |  |  |  |  |  |  | |  |
| I | **.70** | -.46 | **.71** | -.46 | **.69** | **.75** | **.53** | **-.53** | - |  |  |  |  |  | |  |
| J | -.28 | **.54** | -.27 | **.55** | -.27 | -.23 | -.24 | .24 | -.22 | - |  |  |  |  | |  |
| K | **-.68** | **.84** | **-.68** | **.84** | **-.59** | **-.60** | -.45 | .45 | -.46 | .47 | - |  |  |  | |  |
| L | **-.70** | **.92** | **-.70** | **.93** | **-.58** | **-.65** | -.45 | .45 | -.49 | **.52** | **.89** | - |  |  | |  |
| M | .13 | -.12 | .13 | -.11 | .21 | .07 | .28 | -.28 | .01 | -.05 | -.09 | -.04 | - |  | |  |
| N | -.16 | .23 | -.16 | .23 | -.09 | -.11 | .03 | -.03 | -.11 | .10 | .29 | .23 | -.09 | - | |  |
| O | **-.68** | **.68** | **-.68** | **.68** | **-.70** | **-.59** | **-.64** | **.64** | -.45 | .36 | **.60** | **.63** | -.33 | .10 | | - |
| *Note*. A = SIDS, B = UIDS, C = SIDN, D = UIDN, E = ESSD, F = D-max, G = MH Alpha, H = MH Delta, I = SA, J =UA, K = CDIF, L = NCDIF, M = STD, N = RMWSD, O = Pseudo R-squared. *r* > \|.5\| are bolded, \|.3\| < *r* > \|.5\| are underlined. | | | | | | | | | | | | | | |  | |

**References**

Cohen, J. (1988). Statistical power analysis for the behavioral sciences (2nd ed.). Erlbaum.

Cox, D. R., & Snell, E. J. (1989). Analysis of binary data (2nd ed.). Chapman & Hall.

Dorans, N. J., & Holland, P. W. (1993). DIF detection and description: Mantel–Haenszel and standardization. In P. W. Holland & H. Wainer (Eds.), Differential item functioning (pp. 35–66). Erlbaum. <https://doi.org/10.1002/j.2333-8504.1992.tb01440.x>

Dorans, N. J., & Kulick, E. (1983). Assessing unexpected differential item performance of female candidates on SAT and TSWE forms administered in December 1977: An application of the standardization approach. ETS Research Report Series, 1983(1), i–14. <https://doi.org/10.1002/j.2330-8516.1983.tb00009.x>

Dorans, N. J., & Kulick, E. (1986). Demonstrating the utility of the standardization approach to assessing unexpected differential item performance on the Scholastic Aptitude Test. Journal of Educational Measurement, 23(4), 355–368. <https://doi.org/10.1111/j.1745-3984.1986.tb00255.x>

Fidalgo, A. M., Ferreres, D., & Muñiz, J. (2004). Utility of the Mantel–Haenszel procedure for detecting differential item functioning in small samples. Educational and Psychological Measurement, 64(6), 925–936. <https://doi.org/10.1177/0013164404267288>

French, B. F., & Maller, S. J. (2007). Iterative purification and effect size use with logistic regression for differential item functioning detection. Educational and Psychological Measurement, 67(3), 373–393. <https://doi.org/10.1177/0013164406294781>

Gómez-Benito, J., Hidalgo, M. D., & Padilla, J. L. (2009). Efficacy of effect size measures in logistic regression: An application for detecting differential item functioning. Methodology, 5(1), 18–25. <https://doi.org/10.1027/1614-2241.5.1.18>

Hidalgo, M. D., & López-Pina, J. A. (2004). Differential item functioning detection and effect size: A comparison between logistic regression and Mantel–Haenszel procedures. Educational and Psychological Measurement, 64(6), 903–915. <https://doi.org/10.1177/0013164403261769>

Holland, P. W., & Thayer, D. T. (1986). Differential item functioning and the Mantel–Haenszel procedure. ETS Research Report Series, 1986(2), i–24. <https://doi.org/10.1002/j.2330-8516.1986.tb00186.x>

Jodoin, M. G., & Gierl, M. J. (2001). Evaluating type I error and power rates using an effect size measure with the logistic regression procedure for differential item functioning detection. Applied Measurement in Education, 14(4), 329–349. <https://doi.org/10.1207/S15324818AME1404_2>

Li, Z. (2015). A power formula for the Mantel–Haenszel test for differential item functioning. Applied Psychological Measurement, 39(5), 373–388. <https://doi.org/10.1177/0146621614568805>

Liu, I. M., & Agresti, A. (1996). Mantel–Haenszel-type inference for cumulative odds ratios with a stratified ordinal response. Biometrics, 52, 1223–1234. <https://doi.org/10.2307/2532838>

Magis, D., Béland, S., Raîche, G., & Magis, M. D. (2015). difR: Collection of methods to detect DIF in dichotomous and polytomous items (R package).

Mantel, N., & Haenszel, W. (1959). Statistical aspects of the analysis of data from retrospective studies of disease. Journal of the National Cancer Institute, 22(4), 719–748.

McFadden, D. (1974). Conditional logit analysis of qualitative choice behavior. In P. Zarembka (Ed.), Frontiers in econometrics (pp. 105–142). Academic Press.

Meade, A. W. (2010). A taxonomy of effect size measures for the differential functioning of items and scales. Journal of Applied Psychology, 95(4), 728–743. <https://psycnet.apa.org/doi/10.1037/a0018966>

Nagelkerke, N. J. D. (1991). A note on a general definition of the coefficient of determination. Biometrika, 78(3), 691–692.

Penfield, R. D., & Algina, J. (2006). A generalized DIF effect variance estimator for measuring unsigned differential test functioning in mixed-format tests. Journal of Educational Measurement, 43(4), 295–312. <https://doi.org/10.1111/j.1745-3984.2006.00018.x>

Raju, N. S. (1990). Determining the significance of estimated signed and unsigned areas between two item response functions. Applied Psychological Measurement, 14(2), 197–207. <https://doi.org/10.1177/014662169001400208>

Raju, N. S., van der Linden, W. J., & Fleer, P. F. (1995). IRT-based internal measures of differential functioning of items and tests. Applied Psychological Measurement, 19(4), 353–368. <https://doi.org/10.1177/014662169501900405>

Tjur, T. (2009). Coefficients of determination in logistic regression models—A new proposal: The coefficient of discrimination. The American Statistician, 63(4), 366–372. <https://doi.org/10.1198/tast.2009.08210>

Wang, W. C., & Su, Y. H. (2004). Factors influencing the Mantel and generalized Mantel–Haenszel methods for the assessment of differential item functioning in polytomous items. Applied Psychological Measurement, 28(6), 450–480. <https://doi.org/10.1177/0146621604269792>

Zumbo, B. D. (1999). A handbook on the theory and methods of differential item functioning (DIF): Logistic regression modeling as a unitary framework for binary and Likert-type (ordinal) item scores. National Defense Headquarters.

Zwick, R. (2012). A review of ETS differential item functioning assessment procedures: Flagging rules, minimum sample size requirements, and criterion refinement. ETS Research Report Series, 2012(1), i–30. <https://doi.org/10.1002/j.2333-8504.2012.tb02290.x>

Zwick, R., & Ercikan, K. (1989). Analysis of differential item functioning in the NAEP history assessment. Journal of Educational Measurement, 26(1), 55–66. <https://doi.org/10.1111/j.1745-3984.1989.tb00318.x>
